# Use of Herbal Medicines for the Treatment of Mild Mental Disorders and/or Symptoms During Pregnancy: A Cross-Sectional Survey

**DOI:** 10.3389/fphar.2021.729724

**Published:** 2021-10-08

**Authors:** Giulia Gantner, Deborah Spiess, Eliane Randecker, Katharina C. Quack Lötscher, Ana Paula Simões-Wüst

**Affiliations:** Department of Obstetrics, University Hospital Zurich, University of Zurich, Zurich, Switzerland

**Keywords:** pregnancy, herbal medicines, phytopharmacy, mental health disorders, survey, bryophyllum, lavender, valerian

## Abstract

Little is known about the treatment of mild mental disorders and/or symptoms (MDS) during pregnancy. Our main purpose was to compare the use of herbal medicines during pregnancy in women with and without MDS. A questionnaire consisting of 21 multiple-choice questions was distributed in the participating obstetrics clinics or birth centers in the Canton of Zurich, in Switzerland, from August 2018 to March 2019; 398 questionnaires were considered in the analysis. The use of any type of herbal medicines–including pharmaceutical herbal products as well as teas–during pregnancy was reported by 358 women (out of 398, 89.9%). Of these, 272 participants used pharmaceutical herbal products, whereby ginger (49.2%), raspberry leaf (42.7%), bryophyllum (37.8%), chamomile (27.2%), lavender (22%) and iron-rich herbs (12.3%) were the ones most commonly mentioned. More than half (207/398, 52.0%) of all participants reported suffering from MDS during pregnancy; only a few took (synthetic) psychoactive medications (5/398, 1.3%). The percentage of use of pharmaceutical herbal medicines was higher among women reporting MDS than among the remaining women (90.0 vs 75.9%; *p* < 0.001). At the same time, the prevalence of MDS was higher among users of pharmaceutical herbal products than among non-users (59.6 vs 34.0%; *p* = 0.001). Specific questions on candidate herbal medicines for the treatment of mild MDS revealed that bryophyllum (mentioned by 107 women), lavender (56 women) and valerian (20 women) were used to reduce stress, restlessness, sleep disorders and others, in part with perceived good to very good effectiveness and tolerability. The large majority of the pregnant women participating in the survey make use of herbal medicines. The particularly high prevalence of MDS among herbal medicine-users and the very rare use of synthetic psychoactive medications suggest that pregnant women rely on herbal medicines for treatment of mild MDS. The reported good effectiveness and tolerability of a few candidate herbal medicines deserve particular attention.

## Introduction

Little is known about the treatment of mild mental disorders and/or symptoms (MDS) during pregnancy. Most medications for MDS may not only cause side-effects in the mother, but also easily cross the placental barrier and reach the foetus. Concerns on tolerability, teratogenicity and impact on neonatal outcomes exist (Sivojelezova et al., 2005; Rahimi et al., 2006; Grigoriadis et al., 2014; Yonkers et al., 2017; Gao et al., 2018). Pregnant women in need of such medications therefore face a dilemma between using and refraining from synthetic medications.

It is therefore understandable that a considerable proportion of women suffering from mild MDS opt for treatment with herbal medicines, which tend to be perceived as safe (Kalder et al., 2011; Pallivalappila et al., 2013). For instance in Germany, approximately one-fifth of the pregnant women who take herbal medications do it for psychological problems (Munstedt et al., 2013). Even though the toxicity of herbal medications taken during pregnancy has not in most cases been thoroughly investigated, a considerable proportion of health care professionals who deal with pregnant women–midwives, obstetricians, anaesthetists, and especially those with their own experiences–recommend herbal medicine (Stewart et al., 2014). In Switzerland, 40.6% of pregnant women reported using herbal medicine during pregnancy, which was higher than the average proportion detected in a multinational study [average 28.0% (Kennedy et al., 2013)].

Our main purpose was to compare the use of herbal medicines in women with and without MDS. Further goals were to characterise the use, perceived effectiveness and tolerability of a few candidate herbal medicines for mild MDS treatment.

## Methods

### Study Design

The present analysis is based on self-reported data from obstetric patients participating in a cross-sectional survey undertaken between August 2018 and March 2019.

### Ethics Statement

The study was conducted in accordance with the Helsinki Declaration and with Swiss laws and regulations. In compliance with Swiss Federal Law on data protection (Human Research Act, Article 2), since the data were anonymously collected, no special authorisation was needed. This was confirmed by jurisdictional declaration of the ethics committee of Zurich (Enquire BASEC-Nr. Req-2017-00966; letter from December 14, 2017).

### Selection and Description of Participants

The survey took place in the Canton of Zurich–often considered to be representative of the Swiss population as a whole–whose inhabitants correspond to one-sixth of the entire Swiss population. Eight obstetric clinics and birth centres agreed to participate. In these institutions, pregnant women (at or after 28 weeks of pregnancy) or women in the puerperium were invited to participate in the survey if they had not previously completed the questionnaire, could read German, English, French or Italian, and were not emergency patients.

The aim of our survey was to compare the use of herbal medicines during pregnancy in women with and without MDS. Assuming that twice as many pregnant women take herbal medicines for symptoms related to physical conditions than for symptoms related to mental diseases [40 and 20%, respectively, compare with Munstedt et al. (2013)], a sample size of 106 in each group would allow the detection of a difference between the percentage of women taking herbal medicines in the groups with and without MDS. The survey was pursued until 106 women taking (pharmaceutical) herbal medicines in each of the groups with and without MDS had participated.

### Questionnaire

The questionnaire consisted of 21 multiple-choice questions and was available in German, English, French and Italian. Piloting of the questionnaire–distributed to 14 women, at least three per language–was conducted to ensure readability and clarity of the questions.

Four of the 21 (complex) questions were related to the intake of herbal medicines during pregnancy and were inspired by a previous survey (Zuzak et al., 2009). The questionnaire distinguished between herbal infusions/teas and pharmaceutical herbal medicines, but did not specify the type of preparation. For a selection of six herbs, detailed information on perceived effectiveness and tolerability was collected. In the questionnaire, the common names of the herbs were used, with a few exceptions (see Table 1, also for the correspondence between the used names and full taxonomic names).

**TABLE 1:** Herbs mentioned in the questionnaire by common names and corresponding full taxonomical names.

Common names	Full taxonomic names
Mentioned under pharmaceutic products	
Ginger	*Zingiber officinale* Roscoe
Raspberry leaf	*Rubus idaeus* L
Bryophyllum/Goethe plant^a^	*Kalanchoe pinnata* (Lam.) Pers
Chamomile	*Matricaria chamomilla* L^b^
Lavender	*Lavandula angustifolia* Mill
Iron-rich herbs (Floradix^®^)^c^	-
Echinacea	*Echinacea angustifolia* DC
Lemon balm	*Melissa officinalis* L
Valerian	*Valeriana officinalis* L
St. John’s wort	*Hypericum perforatum* L
Horsetail	*Equisetum arvense* L
Passionflower	*Passiflora incarnata* L
Hops	*Humulus lupulus* L
Horse chestnut	*Aesculus hippocastanum* L
Ginseng	*Panax ginseng* C.A. Mey^d^
Golden root (Vitango^®^)^e^	*Rhodiola rosea* L
California poppy	*Escholzia californica* Cham
Winter cherry	*Withania somnifera* (L) Dunal
Kava	*Piper methysticum* G. Forst
Mentioned under teas	
Fennel	*Foeniculum vulgare* Mill
Chamomile	*Matricaria chamomilla* L^b^
Raspberry leaf	*Rubus idaeus* L
Herbal mixture	-
Peppermint	*Mentha × piperita* L
Fruit mixture	-
Lime blossom	*Tilia × europaea* L
Verveine/verbena	*Verbena officinalis* L
Rosehip	*Rosa canina* L
Stinging nettle	*Urtica dioica* L
Lady’s mantle	*Alchemilla alpina* L
Lemon balm	*Melissa officinalis* L
Orange blossom	*Citrus × aurantium* L
Aniseed	*Pimpinella anisum* L
Sage	*Salvia officinalis* L
Elderflower	*Sambucus nigra* L
Cumin	*Cuminum cyminum* L
St. John’s wort	*Hypericum perforatum* L
Ginger	*Zingiber officinale* Roscoe
Horsetail	*Equisetum arvense* L
Valerian	*Valeriana officinalis* L

a Bryophyllum was referred to also by an earlier genus name (that is also a trade name) and one common name, as common names (Goethe plant, life plant, air plant, love plant, and Cathedral bells) are relatively unknown in Switzerland.

b And/or *Chamaemelum nobile* (L.) All.

c Floradix^®^ is a vitamin- and iron-containing food supplement with natural herbal extracts.

d And/or *Panax quinquefolius* L.

e Vitango^®^ is a product based on Golden root (roots and rootstock).

To contextualise our results, data on sociodemographic characteristics, acute/chronic disorders and symptoms and synthetic/conventional medications were collected [in part published elsewhere (Randecker et al., 2020)]. To avoid counting missing answers as “non-use” answers, the questions on medicinal herbal medicines included the option “never used”; in these cases, the number of total answers differs from question to question.

We defined an existing MDS on the basis of specific questions. If the women reported suffering from acute or chronic mental disorder, or related symptoms, or reported taking psychotropic drugs, they were considered to have MDS. No MDS severity assessment was performed; psychotic diseases were not addressed in the questionnaire.

### Data Collection

A total of 1,653 envelopes–each one containing an information sheet, the questionnaire and a post-paid envelope addressed to the Department of Obstetrics, University Hospital Zurich–were handed out to potential participants in obstetric clinics and birth centres in the Canton of Zurich. Professionals were instructed by the study team to distribute the envelopes to patients during prenatal check-ups or during hospitalisation in the early puerperium. Sealing of the envelopes after insertion of the completed questionnaires was emphasised to the patients to ensure anonymity. Data were entered manually into a Microsoft Excel file.

### Statistical Data Analysis

Descriptive statistical analyses were performed using the Statistical Package for the Social Sciences (SPSS), Version 25.0. for Windows (IBM^®^ SPSS^®^ Statistics). Pearson’s chi-square test was used to compare use of herbal medicines between participants with and without MDS, and MDS prevalence between herbal medicines users and non-users. A two-sided *p*-value smaller than 0.05 was considered statistically significant; no correction for multiple testing was applied. The number of missing answers is depicted either in the tables or corresponding legends.

## Results

### Number and Sociodemographic and Health-Related Characteristics of Participants

From a total of 1,653 questionnaires distributed, 398 were completed at or after 28 week’s gestation or shortly after birth and returned either by post or through collection boxes placed in the various institutions (overall response rate: 398/1,653, 24.1%). The 398 participants were treated at the following institutions: University Hospital Zurich (*n* = 164), City Hospital Triemli (*n* = 61), Hospital Zollikerberg (*n* = 50), Hospital Bülach (*n* = 40), Paracelsus-Hospital Richterswil (*n* = 31), Hospital Limmattal (*n* = 21) and Delphys Birthing Center (*n* = 8); in some cases, other institutions (*n* = 10), more than one institution (*n* = 10) or none were named (*n* = 3).

Most women completed the questionnaires in German (362/398, 91.0%), followed by English (28/398, 7.0%), Italian (5/398, 1.3%) and French (3/398, 0.8%). The survey population was comprised mainly of women between 18 and 43 years, with a medium to high level of education (high school to university). The majority of women were from Switzerland (202/398, 50.8%), followed by Germany (64/398, 16.1%) and other European countries (86/398, 21.6%), America (19/398, 4.8%), Asia (16/398, 4.0%), Africa (2/398, 0.5%) and Australia (1/398, 0.3%), others (6/398, 1.5%). The majority of participants had delivered in the days before receiving the questionnaire (221/371, 59.6%); the remaining women were either in pregnancy weeks 28–37 (79/371, 21.3%) or 38-42 (71/371, 19.1%). Women were slightly more often primiparous (193/372, 51.9%) than multiparous. For detailed data, see Supplementary Table S1.

About a quarter of women reported chronic disorders (101/373, 27.1%), of which allergies (29/373, 7.8%), thyroid disorders (24/373, 6.4%), and headaches/migraines (20/373, 5.4%) were the most common (Supplementary Table S2). The following pregnancy-related acute disorders were the most common: 18.6% of women (69/371) reported suffering from gastroesophageal reflux, 17.0% (63/371) from iron deficiency/anemia, and 14.3% (53/371) from morning sickness. The most commonly reported symptoms during pregnancy were fatigue (294/370, 79.5%), nausea (248/365, 67.9%), heartburn (211/359, 58.8%) and shortness of breath (191/359, 53.2%). For additional information on disorders and symptoms during pregnancy, see Supplementary Tables S2, S3, respectively.

### MDS During Pregnancy

**FIGURE 1:**
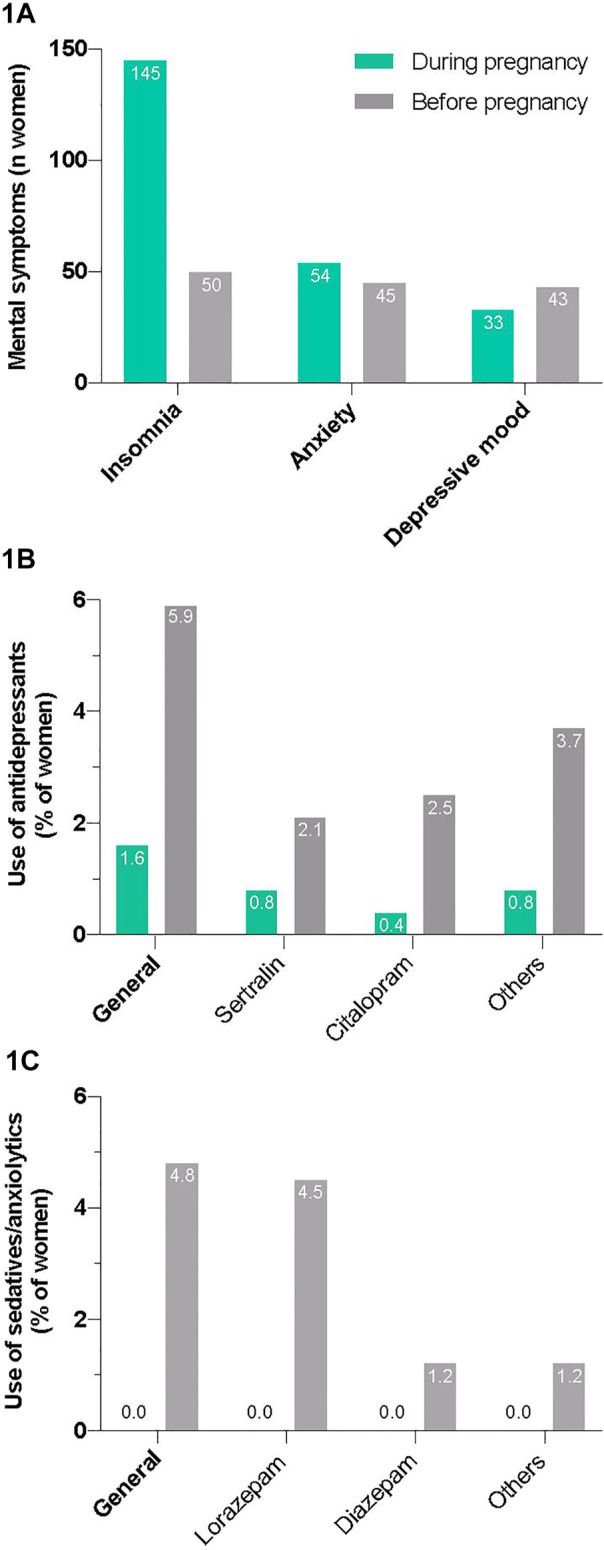
Perceived mental symptoms **(A)** and reported use of antidepressants **(B)** and sedatives/anxiolytics **(C)** during pregnancy and in the time before.

Four percent of the women (15/372) reported one of the following chronic mental disorders: minor depression (*n* = 5); mood disorder (*n* = 5); anxiety disorder (*n* = 3); major depression (*n* = 1) and sleeping disorder (*n* = 1). Moreover, a prevalence of acute mental disorders during pregnancy of 13.2% (48/371) was observed, which included sleeping disorder (*n* = 27); mood disorder (*n* = 13); minor depression (*n* = 5) and anxiety disorder (*n* = 3) (Supplementary Table S2). More than half (51.3%) of the participants reported having suffered from a mental symptom during pregnancy (204/398), namely: insomnia (145/338, 42.9%), anxiety (57/320, 17.8%), and depressive mood (33/335, 9.9%). Compared with the pre-pregnancy period, the prevalence of anxiety and insomnia was higher, whereas the prevalence of depressive mood was slightly lower during pregnancy (Supplementary Table S3). Figure 1A provides an overview of the prevalence of mental symptoms during and before pregnancy.

Only a few participants used antidepressants (5/315, 1.6%) and none used sedatives/anxiolytics during pregnancy (Figures 1B,C; Supplementary Table S4). The number of participants taking antidepressants and sedatives/anxiolytics before becoming pregnant was markedly higher (19/321 or 5.9%; and 15/315 or 4.8%, respectively). Two women (2/240; 0.8%) answered that they were taking an antidepressant not mentioned in the questionnaire during pregnancy, namely escitalopram (*n* = 1) and paroxetine (*n* = 1), both selective serotonin reuptake inhibitors (SSRIs); additional other sedatives/anxiolytics were not mentioned.

### Use of Herbal Medicines in General and Versus MDS

The vast majority of women reported using some type of herbal medications, either as teas or as pharmaceutic/clinical medicines, during pregnancy (358/398, 89.9%). Of these, 272 participants (272/325, 83.7%) used pharmaceutical herbal products, most frequently ginger (49.2%), raspberry leaf (42.7%), bryophyllum (37.8%), chamomile (27.2%), lavender (22.0%) and iron-rich herbs (12.3%); see Table 2. The majority of participants (263/358, 86.5%) who reported using herbal medicines during pregnancy, also used herbal medicines before the current pregnancy. The consumption of teas was very widespread in our study population (Table 3).

**TABLE 2:** Use of pharmaceutical herbal medicines during pregnancy by participants with and without mental disorders and/or symptoms (MDS).

Medicine	Total users (*n* = 272)	With MDS (*n* = 162)	Without MDS (*n* = 110)	*p*-value
	n/N*(%)^a^	n/N*(%)^a^	n/N*(%)^a^	
Ginger	121/246 (49.2)	71/147 (48.3)	50/99 (50.5)	0.734
Raspberry leaf	106/248 (42.7)	58/148 (39.2)	48/100 (48.0)	0.169
Bryophyllum	93/246 (37.8)	75/148 (50.7)	18/98 (18.4)	<0.001
Chamomile	66/243 (27.2)	40/144 (27.8)	26/99 (26.3)	0.794
Lavender	52/236 (22.0)	44/141 (31.2)	8/95 (8.4)	<0.001
Iron-rich herbs	30/243 (12.3)	19/144 (13.2)	11/99 (11.1)	0.628
Echinacea	19/232 (8.2)	13/138 (9.4)	6/94 (6.4)	0.408
Lemon balm	19/232 (8.2)	9/137 (6.6)	10/95 (10.5)	0.280
Valerian	11/234 (4.7)	10/140 (7.1)	1/94 (1.1)	0.031
St. John’s wort	7/231 (3.0)	6/138 (4.3)	1/93 (1.1)	0.155
Horsetail	7/235 (3.0)	5/141 (3.5)	2/94 (2.1)	0.531
Passionflower	5/235 (2.1)	5/140 (3.6)	0/95 (0.0)	0.063
Hops	4/231 (1.7)	4/137 (2.9)	0/94 (0.0)	0.095
Horse chestnut	4/229 (1.7)	3/135 (2.2)	1/94 (1.1)	0.510
Ginseng	4/231 (1.7)	1/137 (0.7)	3/94 (3.2)	0.159
Golden root	3/233 (1.3)	3/139 (2.2)	0/94 (0.0)	0.152
Others	23/113 (20.4)	13/72 (18.1)	10/41 (24.4)	0.421

^a^Percentage values without considering missing data.

Data are sorted by frequency of total herbal medicine users. The questionnaire also contained items on California poppy, winter cherry and kava; no participant reported their use.

**TABLE 3:** The most commonly used teas during pregnancy considering mental disorders and/or symptoms (MDS).

Tea	Total users (*n* = 329)	With MDS (*n* = 172)	Without MDS (*n* = 157)	*p*-value
	n/N*(%)^a^	n/N*(%)^a^	n/N*(%)^a^	
Fennel	157/329 (47.7)	87/172 (50.6)	70/157 (44.6)	0.301
Chamomile	153/329 (46.5)	82/172 (47.7)	71/157 (45.2)	0.621
Raspberry leaf	150/329 (45.6)	76/172 (44.2)	74/157 (47.1)	0.552
Herbal mixture	130/329 (39.5)	69/172 (40.1)	61/157 (38.9)	0.782
Peppermint	122/329 (37.1)	70/172 (40.7)	52/157 (33.1)	0.175
Fruit mixture	104/329 (31.6)	56/172 (32.6)	48/157 (30.6)	0.672
Lime blossom	68/329 (20.7)	42/172 (24.4)	26/157 (16.6)	0.096
Verveine/verbena	62/329 (18.8)	31/172 (18.0)	31/157 (19.7)	0.609
Rosehip	58/329 (17.6)	36/172 (20.9)	22/157 (14.0)	0.095
Stinging nettle	55/329 (16.7)	25/172 (14.5)	30/157 (19.1)	0.216
Lady’s mantle	38/329 (11.6)	24/172 (14.0)	14/157 (8.9)	0.148
Lemon balm	37/329 (11.2)	17/172 (9.9)	20/157 (12.7)	0.328
Orange blossom	36/329 (10.9)	20/172 (11.6)	16/157 (10.2)	0.663
Aniseed	28/329 (8.5)	17/172 (9.9)	11/157 (7.0)	0.439
Sage	26/329 (7.9)	17/172 (9.9)	9/157 (5.7)	0.159
Elderflower	23/329 (7.0)	12/172 (7.0)	11/157 (7.0)	0.997
Cumin	23/329 (7.0)	15/172 (8.7)	8/157 (5.1)	0.193
St. John’s wort	14/329 (4.3)	7/172 (4.1)	7/157 (4.5)	0.870
Ginger	11/329 (3.3)	4/172 (2.3)	7/157 (4.5)	0.287
Horsetail	8/329 (2.4)	6/172 (3.5)	2/157 (1.3)	0.190
Valerian	3/329 (0.9)	2/172 (1.2)	1/157 (0.6)	0.613
Others	30/329 (9.1)	18/172 (10.5)	12/157 (7.6)	0.366

^a^Percentage values without considering missing data.

Data are sorted by frequency of use of teas by total population.

**FIGURE 2:**
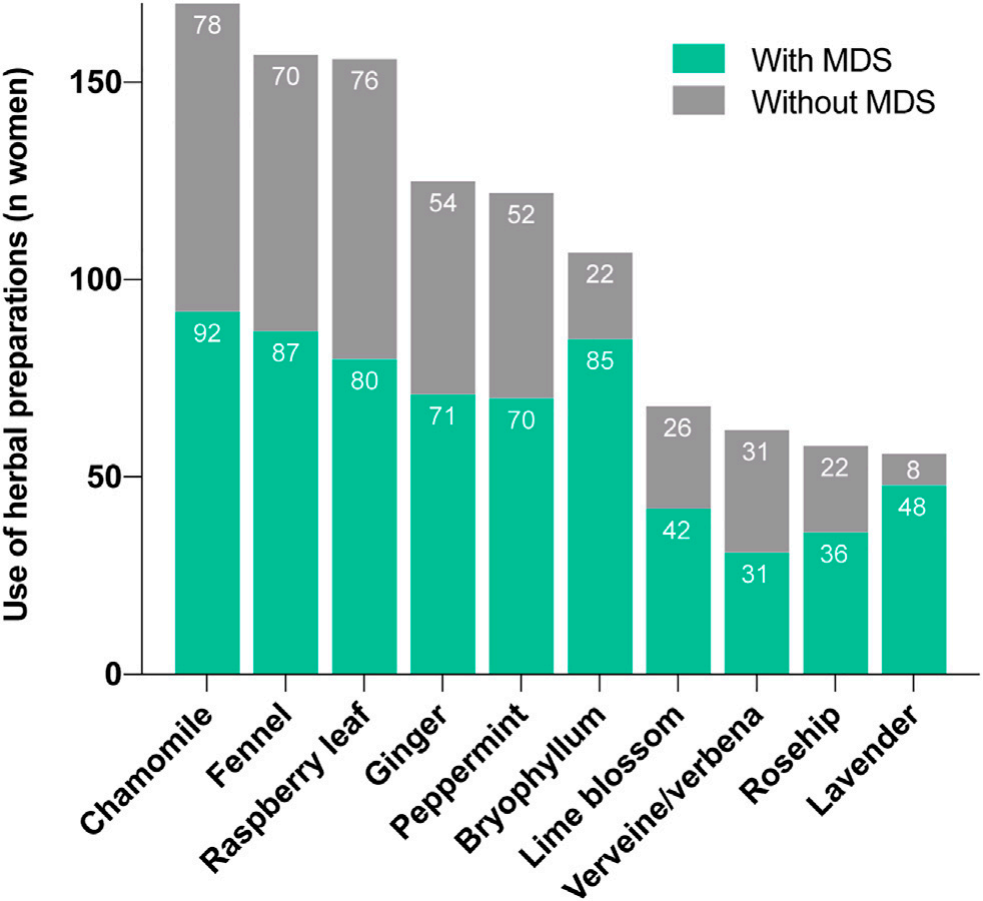
Comparison of the most frequently used herbal medicines (pharmaceutical products and teas) in women with and without mental disorders and/or symptoms (MDS).

The percentage of use of pharmaceutical herbal medicines was higher among women reporting MDS than among the remaining women (teas not considered; 162/180, 90.0% vs 110/145, 75.9%; *p* < 0.001). Almost 60% of the women who used pharmaceutical herbal medicines suffered from MDS (162/272, 59.6%), which is significantly higher than the corresponding value for non-users (18/53, 34.0%; *p* = 0.001). More than half of the women who used any type of herbal preparation, teas and pharmaceutical herbal medicines, reported MDS (192/358, 53.6%). Figure 2 shows the most frequently used herbal medicines, pharmaceutical herbal medicines and teas counted together. Several differences between the groups with and without MDS are apparent. Pregnant women with MDS also more often reported using medicines from integrative and complementary medicine than women without (Table 4).

**TABLE 4:** Use of medicines from integrative and complementary medicine during pregnancy.

	Total users (*n* = 116)	With MDS (*n* = 78)	Without MDS (*n* = 38)	*p*-value
	n/N*(%)^a^	n/N*(%)^a^	n/N*(%)^a^	
Anthroposophic medicine	73/326 (22.4)	51/78 (65.4)	22/38 (57.9)	0.759
Herbal medicines	72/336 (21.4)	51/78 (65.4)	21/38 (55.3)	0.600
Others	17/303 (5.6)	11/78 (14.1)	6/38 (15.8)	0.778
Not sure about intake	30/364 (8.2)	4/78 (5.1)	5/38 (13.2)	0.131
Homeopathy	61/330 (18.5)	40/78 (51.3)	21/38 (55.3)	0.418
Mother tinctures	19/352 (5.4)	14/78 (17.9)	5/38 (13.2)	0.579
Diluted herbal components	33/351 (9.4)	24/78 (30.8)	9/38 (23.7)	0.572
Others	28/320 (8.8)	16/78 (20.5)	12/38 (31.6)	0.259
Not sure about intake	18/370 (4.9)	13/78 (7.7)	2/38 (5.3)	0.684
Traditional Chinese medicine	24/336 (7.1)	17/78 (21.8)	7/38 (18.4)	0.735
Herbal medicines	21/353 (5.9)	16/78 (20.5)	5/38 (13.2)	0.331
Others	5/329 (1.5)	2/78 (2.6)	3/38 (7.9)	0.193
Not sure about intake	14/367 (3.8)	5/78 (6.4)	1/38 (2.6)	0.380
Ayurvedic medicine	12/337 (3.6)	8/78 (10.3)	4/38 (10.5)	0.910
Herbal medicines	12/351 (3.4)	8/78 (10.3)	4/38 (10.5)	0.959
Others	1/328 (0.3)	1/78 (1.3)	0/38 (0.0)	0.493
Not sure about intake	17/367 (4.6)	6/78 (7.7)	1/38 (2.6)	0.313

^a^Percentage values without considering missing data.

### Effectiveness and Tolerability of Candidate Herbal Medicines for MDS Treatment

Some questions on the questionnaire specifically targeted the use of well-known plants for the treatment of mild MDS, namely St. John’s wort, hops, valerian, lavender, and bryophyllum.

**FIGURE 3:**
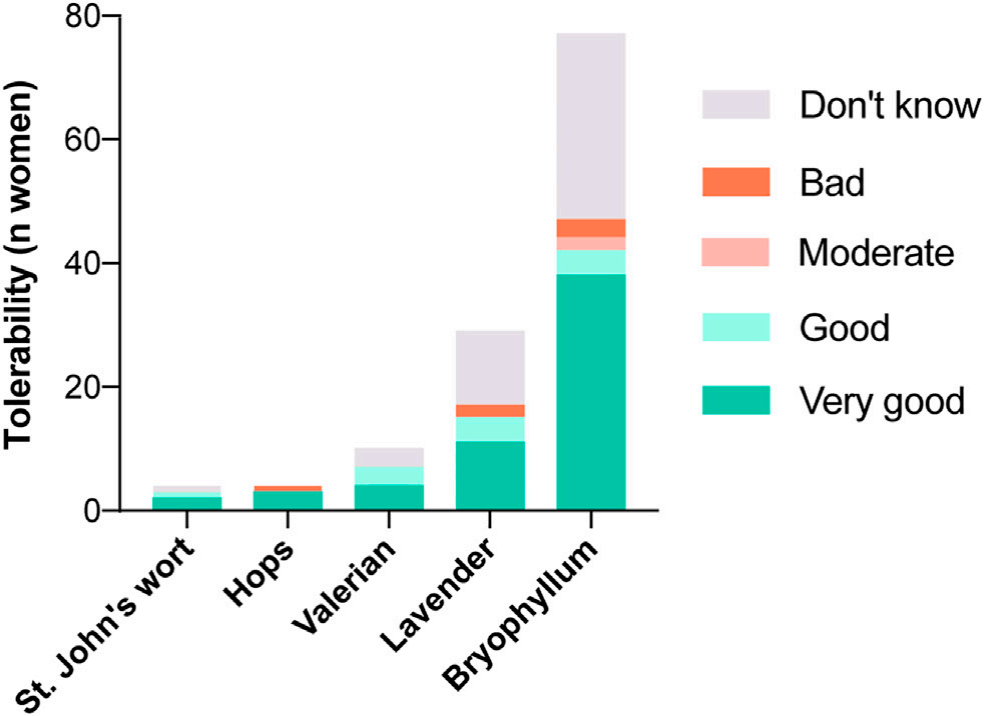
Experiences with candidate herbal medicines for the treatment of mental disorders and/or symptoms (MDS) during pregnancy regarding their tolerability.

St. John’s wort was taken by 3.5% of women who answered the corresponding questions (12/341), in half of the cases during pregnancy weeks 28–42. Three women reported good to very good tolerability (Figure 3) and moderate to good effectiveness. St. John’s wort was mostly taken for the treatment of mood disorders (4/12, 33.3%).

Hops was used by 2.3% of women (8/343), in all phases of pregnancy. Hops was tolerated well: three of eight women reported very good tolerability and one of eight reported poor tolerability. Seven of eight women evaluated its effectiveness as very good. The two most frequently reported indications were stress and restlessness (both 3/8, 37.5%), followed by sleep disorders (2/8, 25.0%).

**FIGURE 4:**
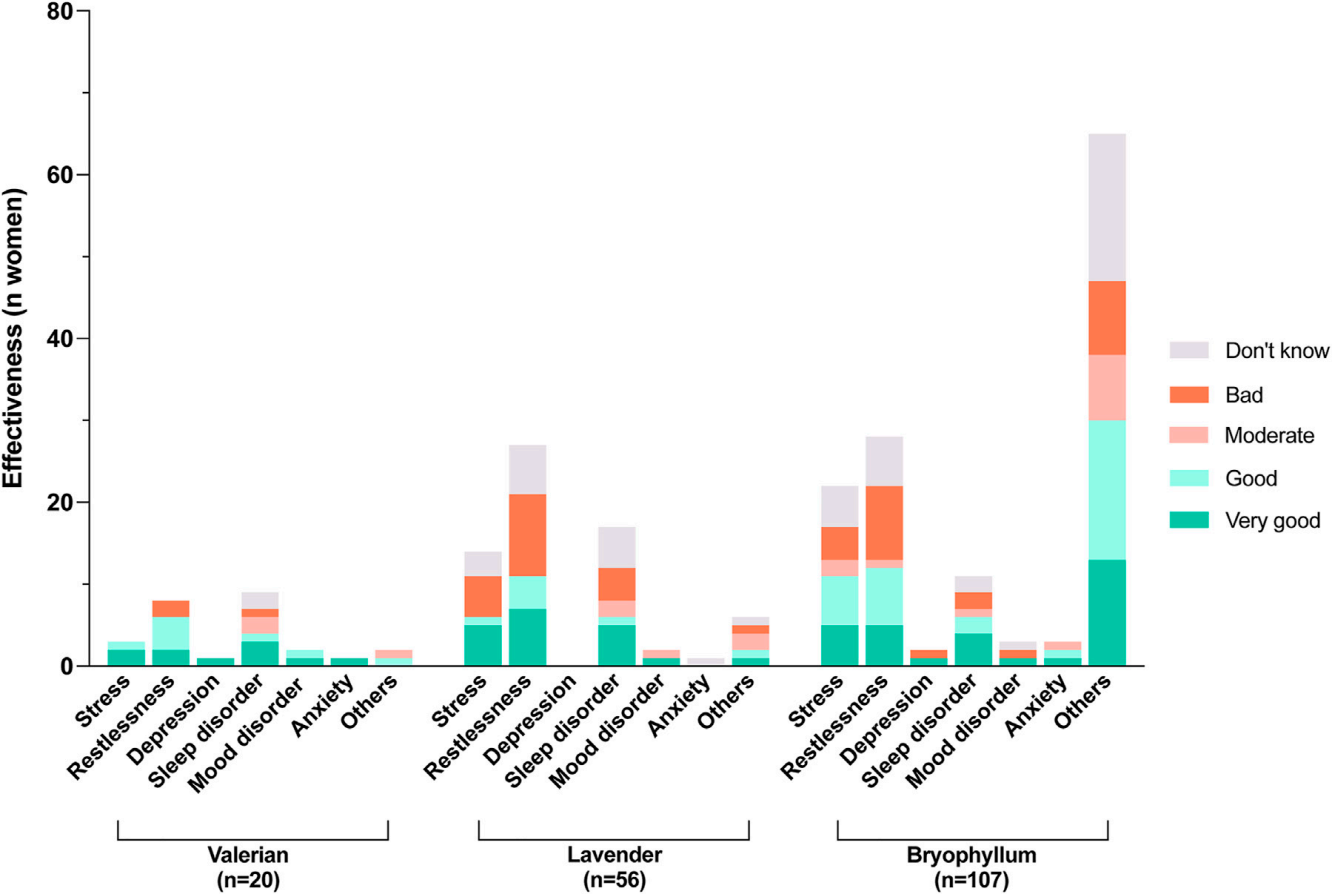
Experiences with candidate herbal medicines for the treatment of mental disorders and/or symptoms (MDS) during pregnancy regarding their effectiveness.

Valerian was used most frequently in the last trimester (7/20, 35.0%). Overall, 20 women reported using valerian (20/344, 5.8%), none of the participants reported poor tolerability and the majority tolerated the herb well (“very good” 4/20, 20.0%; “good” 3/20, 15.0%). As shown in Figure 4, half of the participants rated its effectiveness as very good (10/20, 50.0%). Valerian was used most commonly to overcome sleep disorders (9/20, 45.0%), and restlessness (8/20, 40.0%).

The use of lavender was also increased toward the end of pregnancy. Lavender was used by 16.2% of women (56/345) and 11 of 56 women rated its tolerability as very good. Its effectiveness was described as good to very good by almost half of the women who used it (26/56, 46.4%), but bad by 35.7% of women (20/56). Lavender was used mostly to combat restlessness (28/56, 50.0%), sleep disorders (19/56, 33.9%) and stress (15/56, 26.8%).

Bryophyllum was used during pregnancy by almost one-third of the participants (107/360, 29.7%). Of these, more than three-quarters used it during pregnancy weeks 28–42 (84/107, 78.5%). Only a few women (3/107) reported poor tolerability. Regarding the effectiveness of bryophyllum, 58.8% of women rated it good to very good (63/107, all indications together; comparable percentages in the main single indications; see Figure 4). Restlessness was mentioned as a main indication by 27.1% of women (29/107) and 22.4% (24/107) used bryophyllum for stress relief. Another 10.3% (11/107) reported using bryophyllum for sleep disorders. Sixty-six percent of the women who had used bryophyllum (71/107, 66.4%) reported taking the herbal medicine for other reasons than those listed, and the majority wrote that the indications were any type of contractions (44/107, 41.1%). In addition, some women reported using bryophyllum for uterus soothing (*n* = 3), against abdominal cramps/pain (*n* = 3), against general pain (*n* = 1) and against cervix shortening (*n* = 1).

## Discussion

### Main Findings

In our study population, the majority of women made use of herbal medicines, including pharmaceutical herbal products and teas. A wide variety of herbal products were mentioned by the survey participants, but ginger, raspberry leaf, bryophyllum, chamomile, lavender and iron-rich herbs were most commonly used. Our data further show that more than half (52.0%) of all participants had MDS during pregnancy, although few made use of (synthetic) psychoactive medications. The prevalence of MDS was higher among users of pharmaceutical herbal medicines than among non-users. Focusing on the most commonly used herbal medicines, users of bryophyllum and lavender reported suffering from MDS particularly often. Specific questions about candidate herbal medicines for the treatment of MDS revealed that bryophyllum (mentioned by 107 women), lavender (56 women) and valerian (20 women) were used to reduce stress, restlessness, sleep disorders and others, frequently with perceived good to very good effectiveness and tolerability.

### Strengths and Limitations

All data were self-reported. While the number of pregnant participants (*n* = 398) can be considered a strength of the present survey, the moderate response rate of 24.1% is a limitation of the study. To avoid selections biases, following measures were taken: 1) all hospitals from the Canton of Zurich with an obstetrics ward were invited to participate; 2) the institutions that agreed to participate were regularly reminded of the survey; 3) the questionnaires were available in four languages (the three main country languages and English); 4) mental conditions were not mentioned in the title or cover letter of the questionnaire; 5) it was clearly stated that the survey was fully anonymous. Nevertheless, some selection bias due to interest in herbal medicines (mentioned in the title and cover letter) or the limited number of available questionnaire languages cannot be excluded. In the case of patients from the University Hospital Zurich (41.2% of survey participants), an internal analysis of the main patient demographic data revealed comparable characteristics to those of the survey participants (own unpublished observations). Finally, most questionnaires were handed over to women after delivery. While this can be considered a strength–as postpartum women are able to describe their use of herbal medicines throughout the whole pregnancy–it is conceivable that some participants may not have remembered the medicines taken at the beginning of pregnancy.

### Use of Pharmaceutic Herbal Medicines During Pregnancy

The high use of herbal medicines observed in the present survey is consistent with findings of a previous pilot survey conducted in a comparable population in the late 1990s (Gut et al., 2004). Studies from other countries reported lower use, and a multinational study of the use of herbal medications in pregnancy in 23 countries, and involving 9,459 women, revealed a markedly lower rate [28.9% of the women (Kennedy et al., 2013)]. This could be related to different perceptions of “herbal medicines” among participants in the different studies. In general, women in our study seemed to be more likely to use herbal medicines if they were primiparous and had used herbal medicines in the past/before pregnancy (data not shown). This could also be seen in an Australian study (Low Dog, 2009), where the most commonly used herbals were ginger, cranberry, valerian, raspberry, chamomile and peppermint (Kennedy et al., 2013), which is similar to our results.

In the following, we will focus on the herbal medicines used by at least 20% of the participants, first with respect to frequency of use during pregnancy and effectiveness, then summarising what is known on their safety. Our finding that ginger was the most commonly used herbal medicine confirms the results of previous surveys, and corresponds well with the high prevalence of nausea during pregnancy found in our survey (67.9%). Ginger can be considered a possibly effective treatment for nausea and vomiting during pregnancy (Viljoen et al., 2014). Raspberry leaf was the second most commonly used herbal medicine among pregnant women in our study, in accordance with studies showing that it is often recommended by midwives (Allaire et al., 2000; Holst et al., 2009). It is used to strengthen or prepare the uterus, to soften the cervix, and to induce and shorten labor (Briggs et al., 2022). In a placebo-controlled randomized trial, raspberry leaf tablets did not shorten the first stage of labor, but resulted in a small shortening of the second stage and less forceps deliveries (Simpson et al., 2001). Bryophyllum was the third most commonly used herbal medicine. Because this plant is recommended in several perinatal centres in Switzerland for the treatment of anxiety states, restlessness, and sleep disturbances (Schenkel et al., 2018), the questionnaire contained additional questions about its use (see below). Nevertheless, most women used bryophyllum for the treatment of other disorders, often related to the attenuation of uterine contractions. In anthroposophic medicine, bryophyllum was introduced in the 1970s as a well-tolerated agent for the treatment of preterm labor (Fürer et al., 2016; Hamburger et al., 2017); in Switzerland it is recommended for this indication (Schenkel et al., 2018) and commonly used in the main perinatal institutions (Fürer et al., 2015). Chamomile, the fourth most commonly used medicine has been also widely used and is known as a treatment for nausea and vomiting during pregnancy (Sanaati et al., 2016). Finally, lavender was frequently used (see below for information on its anxiolytic effects).

The Committee on Herbal Medicinal Products (CHMP) of the European Medicine Agency (EMA) does not recommend the use of ginger (European Medicines Agency, 2012b), raspberry leaf (European Medicines Agency, 2014), chamomile (European Medicines Agency, 2015) and lavender (European Medicines Agency, 2012a) during pregnancy because of insufficient safety data; so far, no community herbal monograph was published on bryophyllum. According to a reference work on drugs in pregnancy (Briggs et al., 2022), ginger and raspberry leaf are classified as compatible with pregnancy, whereas about chamomile it is considered that human data are limited and no relevant human data are available. According to a systematic review and meta-analysis from 2014, ginger can be considered a harmless treatment for nausea and vomiting during pregnancy: here no significant differences were found between the ginger and placebo treated groups for all reported adverse effects in various studies (Viljoen et al., 2014). A systematic review come to comparable conclusions (Stanisiere et al., 2018). With respect to raspberry leaf use, an unclear association with caesarean sections (Nordeng et al., 2011) and an interaction with a conventional medicine (hypoglycaemia when used with insulin) have been reported (Cheang et al., 2016). A randomized trial, however, revealed no adverse effects for mother or child (Simpson et al., 2001). Several retrospective and prospective studies on the use of bryophyllum during pregnancy are indicative of a good safety profile (Lambrigger-Steiner et al., 2014; Fürer et al., 2015; Fürer et al., 2016; Hamburger et al., 2017; Simões-Wüst et al., 2018). A qualitative study pointed towards an association between regular use of chamomile during pregnancy and a higher incidence of threatening miscarriages and preterm labor [without correction for possible confounders; Cuzzolin et al. (2010)].

### Herbal Medicines and MDS Treatment

Of the herbal medicines specifically addressed in the present questionnaire, there were several that–irrespective of pregnancy–are used in the treatment of mild MDS. Evidence for their use during pregnancy is still scarce, therefore the existing studies are briefly discussed below (on the herbal medicines used by at least 20 participants).

**Valerian** was mostly used to treat restlessness and sleep disorders. As a sleep-aid, its benefits and side effect profile have been shown in several studies (Dorn, 2000; Andreatini et al., 2002; Hattesohl et al., 2008). In a small survey conducted in southern Italy, no influence of valerian use on pregnancy and neonatal outcomes was found (*n* = 9) (Low Dog, 2009). Data from the Swedish Birth Register from 1995 to 2004 also suggest good safety, as no abnormalities were found in the infants of mothers who had taken phytotherapeutics–often valerian–during pregnancy (*n* = 787, 0.9% of all mothers in the register).

In our study, women reported using **lavender** more often during the last trimester of pregnancy, mainly against restlessness, sleep disorders and stress. Several clinical trials revealed good efficacy and tolerability of a medicine prepared from lavender flowers in the treatment of anxiety (Kasper et al., 2010; Woelk and Schlafke, 2010; Kasper et al., 2014). Furthermore, lavender tea has been shown to enhance the effects of the antidepressant citalopram (Nikfarjam et al., 2013). We are aware of only one previous study during pregnancy: in a randomised placebo-controlled trial, lavender cream was shown to reduce anxiety, stress and depression (Effati-Daryani et al., 2015).

Although most participants used **bryophyllum** to treat indications other than MDS, this plant was also used to treat restlessness, stress, sleep disorders, mood disorders, anxiety, and depression. In anthroposophic medicine, the use of bryophyllum medicines for mental disorders is well documented (Simões-Wüst et al., 2012). Prospective observational studies revealed improvements in sleep quality after treatment with bryophyllum in pregnant women (Lambrigger-Steiner et al., 2014) [and in cancer patients (Simões-Wüst et al., 2015)].

The particularly high prevalence of MDS among herbal medicine-users and the rare use of synthetic psychoactive medications suggest that pregnant women avoid them and prefer recurring to herbal medicines for mild MDS treatment. The reported positive experiences with some candidate herbal medicines for mild MDS treatment and their well-perceived tolerability deserve particular attention.

## Data Availability

The raw data supporting the conclusions of this article will be made available by the authors, without undue reservation.
