# Supplementary material for: Use of Herbal Medicines for the Treatment of Mild Mental Disorders and/or Symptoms During Pregnancy: A Cross-Sectional Survey
Source: Front Pharmacol. 2021 Oct 8;12:729724. doi: 10.3389/fphar.2021.729724 (PMC8531499; doi:10.3389/fphar.2021.729724)
Supplement: Supplementary file 1 [file DataSheet1.docx]

**SUPPLEMENTARY INFORMATION**

**Use of herbal medicines for the treatment of mild mental disorders and/or symptoms during pregnancy: a cross-sectional survey**

G. Gantner, D. Spiess, E. Randecker, K.C. Quack Lötscher, A.P. Simões-Wüst

Department of Obstetrics, University Hospital Zurich, University of Zurich, 8091 Zurich, Switzerland

Keywords: pregnancy, mental health disorders, phytopharmacy, behaviour, survey

| **Suppl. Table 1** General and pregnancy-related characteristics. | | | | |
| --- | --- | --- | --- | --- |
|  | **All participants^a^** | **Herbal preparation users** | | |
|  |  | **Total** | **With MDS** | **Without MDS** |
| n/% | 398/100 | 358/100 | 192/100 | 166/100 |
| **Age (years)** |  |  |  |  |
| 18-22 | 3/0.8 | 3/0.8 | 2/1.0 | 1/0.6 |
| 23-27 | 27/6.8 | 23/6.4 | 11/5.7 | 12/7.3 |
| 28-32 | 129/32.6 | 114/31.9 | 59/30.7 | 55/33.3 |
| 33-37 | 156/39.4 | 139/38.9 | 76/39.6 | 63/38.2 |
| 38-42 | 79/19.9 | 76/21.3 | 42/21.9 | 34/20.6 |
| >43 | 2/0.5 | 2/0.6 | 2/1.0 | 0/0.0 |
| Missing | 2/- | 1/- | 0/- | 1/- |
| **Health insurance** |  |  |  |  |
| Basic | 117/29.5 | 102/28.6 | 59/30.7 | 43/26.1 |
| Basic+supplementary | 195/49.2 | 176/49.3 | 95/49.5 | 81/49.1 |
| Semi-private | 61/15.4 | 58/16.2 | 28/14.6 | 30/18.2 |
| Private | 23/5.8 | 21/5.9 | 10/5.2 | 11/6.7 |
| Missing | 2/(0.5) | 1/- | 0/- | 1/- |
| **Marital status** |  |  |  |  |
| Married | 273/68.9 | 242/67.8 | 120/62.5 | 122/73.9 |
| Cohabitant | 81/20.5 | 75/21.0 | 41/21.4 | 34/20.6 |
| Single | 41/10.4 | 39/10.9 | 30/15.6 | 9/5.5 |
| Divorced/separated | 1/0.3 | 1/0.3 | 1/0.5 | 0/0.0 |
| Missing | 2/- | 1/- | 0/- | 1/- |
| **Place of birth** |  |  |  |  |
| Switzerland | 202/50.8 | 184/51.5 | 97/49.7 | 87/52.7 |
| Germany | 64/16.1 | 60/16.8 | 31/21.2 | 23/13.9 |
| Other countries in Europe | 86/21.6 | 71/19.8 | 37/19.3 | 34/20.6 |
| America | 19/4.8 | 18/5.0 | 7/3.7 | 11/6.6 |
| Africa | 2/0.5 | 2/0.6 | 1/0.5 | 1/0.6 |
| Asia | 16/4.0 | 15/4.2 | 8/4.2 | 7/4.2 |
| Australia | 1/0.3 | 1/0.3 | 1/0.5 | 0/0.0 |
| Others | 6/1.5 | 6/1.7 | 4/2.1 | 2/1.2 |
| Missing | 2/- | 1/- | 0/- | 1/- |
| **School education** |  |  |  |  |
| No school qualification | 1/0.3 | 1/0.3 | 0/0.0 | 1/0.6 |
| Secondary school | 9/2.3 | 6/1.7 | 3/1.6 | 3/1.8 |
| Professional apprenticeship | 68/17.2 | 54/15.1 | 32/16.7 | 22/13.3 |
| High school | 23/5.8 | 20/5.6 | 12/6.3 | 8/4.8 |
| Higher professional school | 101/25.4 | 96/26.9 | 51/26.6 | 45/27.3 |
| University | 189/47.5 | 175/49.0 | 90/46.9 | 85/51.5 |
| Others | 5/1.3 | 5/1.4 | 4/2.1 | 1/0.6 |
| Missing | 2/- | 1/- | 0/- | 1/- |
| **Work situation at the beginning of pregnancy** |  |  |  |  |
| Employed in the health care sector | 93/23.8 | 85/24.1 | 44/23.0 | 41/25.3 |
| Employed in another sector | 242/61.9 | 217/61.5 | 116/60.7 | 101/62.3 |
| Housewife | 23/5.9 | 20/5.7 | 10/5.2 | 10/6.2 |
| Student | 7/1.8 | 7/2.0 | 4/2.1 | 3/1.9 |
| Self-employed or none of the above | 19/4.8 | 18/5.1 | 14/7.3 | 4/2.5 |
| Job seeker | 7/1.8 | 6/1.7 | 3/1.6 | 3/1.9 |
| Missing | 7/- | 5/- | 1/- | 4/- |
| **Monthly gross income of household (CHF)** |  |  |  |  |
| <4’000 | 56/14.5 | 47/13.5 | 32/17.0 | 15/9.3 |
| 4’000-8’000 | 150/38.8 | 134/38.4 | 67/35.6 | 67/41.6 |
| 8’000-12’000 | 94/24.3 | 88/25.2 | 44/23.4 | 44/27.3 |
| >12’000 | 86/22.3 | 80/22.9 | 45/23.9 | 35/21.8 |
| Missing | 12/- | 9/- | 4/- | 5/- |
| **Hospital** |  |  |  |  |
| University Hospital Zurich | 164/41.5 | 147/41.3 | 82/42.7 | 65/39.6 |
| City Hospital Triemli | 61/15.4 | 55/15.4 | 28/14.6 | 27/16.5 |
| Hospital Limmattal | 21/5.3 | 18/5.1 | 6/3.1 | 12/7.3 |
| Hospital Zollikerberg | 50/12.7 | 47/13.2 | 27/14.1 | 20/12.2 |
| Hospital Bülach | 40/10.1 | 32/9.0 | 13/6.8 | 19/11.6 |
| Paracelsus-Hospital Richterswil | 31/7.8 | 31/8.7 | 19/9.9 | 12/7.3 |
| Delphys Birthing Center | 8/2.0 | 8/2.2 | 6/3.1 | 2/1.2 |
| Others | 10/2.5 | 9/2.5 | 5/2.6 | 4/2.4 |
| Multiple | 10/2.5 | 9/2.5 | 6/3.1 | 3/1.8 |
| Missing | 3/- | 2/- | 0/- | 2/- |
| **Language of the survey** |  |  |  |  |
| German | 362/91.0 | 328/91.6 | 180/93.8 | 148/89.2 |
| English | 28/7.0 | 22/6.1 | 7/3.6 | 15/9.0 |
| French | 3/0.8 | 3/0.8 | 1/0.5 | 2/1.2 |
| Italian | 5/1.3 | 5/1.4 | 4/2.1 | 1/0.6 |
| **Pregnancy week** |  |  |  |  |
| 28-37 | 79/21.3 | 75/22.4 | 51/28.3 | 24/15.5 |
| 38-42 | 71/19.1 | 65/19.4 | 36/20.0 | 29/18.7 |
| Postpartum period | 221/59.6 | 195/58.2 | 93/51.7 | 102/65.8 |
| Missing | 27/(6.8) | 23/(6.4) | 12/(6.3) | 11/(6.6) |
| **First pregnancy** |  |  |  |  |
| Yes | 193/51.9 | 177/52.7 | 100/55.6 | 77/49.4 |
| No | 179/48.1 | 159/47.3 | 80/44.4 | 79/50.6 |
| Missing | 26/- | 22/- | 12/- | 10/- |
| **Number of previous pregnancies** |  |  |  |  |
| 0 | 191/52.3 | 175/53.0 | 98/55.4 | 77/50.3 |
| 1 | 66/18.1 | 62/18.8 | 32/18.1 | 30/19.6 |
| 2 | 65/17.8 | 53/16.1 | 26/14.7 | 27/17.6 |
| 3 | 30/8.2 | 27/8.2 | 11/6.2 | 16/10.5 |
| 4 | 7/1.9 | 7/2.1 | 5/2.8 | 2/1.3 |
| 5 | 4/1.1 | 4/1.2 | 3/1.7 | 1/0.7 |
| 6 | 2/0.5 | 2/0.6 | 2/1.1 | 0/0.0 |
| Missing | 33/- | 28/- | 15/- | 13/- |
| **First child** |  |  |  |  |
| Yes | 204/55.4 | 188/56.5 | 102/57.3 | 86/55.5 |
| No | 164/44.6 | 145/43.5 | 76/42.7 | 69/44.5 |
| Missing | 30/- | 25/- | 14/- | 11/- |
| **Recreational drug consumption in pregnancy** |  |  |  |  |
| **Low alcohol consumption** (<2 drinks/week) |  |  |  |  |
| Yes | 15/4.1 | 13/3.9 | 9/5.1 | 4/2.6 |
| No | 351/95.9 | 317/96.1 | 169/94.9 | 148/97.4 |
| Missing | 32/- | 28/- | 14/- | 14/ |
| **Moderate alcohol consumption** (2-4 drinks/week) | |  |  |  |
| Yes | 0/0.0 | 0/0.0 | 0/0.0 | 0/0.0 |
| No | 366/100.0 | 330/100.0 | 178/100.0 | 152/100.0 |
| Missing | 32/- | 28/- | 14/- | 14/- |
| **Severe alcohol consumption** (>5 drinks/week) | |  |  |  |
| Yes | 1/0.3 | 1/0.3 | 1/0.6 | 0/0.0 |
| No | 365/99.7 | 329/99.7 | 177/99.4 | 152/100.0 |
| Missing | 32/- | 28/- | 14/- | 14/- |
| **Smoking** |  |  |  |  |
| Yes | 15/4.1 | 15/4.5 | 7/3.9 | 8/5.3 |
| No | 351/95.9 | 315/95.5 | 171/96.1 | 144/94.7 |
| Missing | 32/- | 28/- | 14/- | 14/- |
| **Cannabis** |  |  |  |  |
| Yes | 3/0.8 | 3/0.9 | 3/1.7 | 0/0.0 |
| No | 363/99.2 | 327/99.1 | 175/98.3 | 152/100.0 |
| Missing | 32/- | 28/- | 14/- | 14/- |
| **Cocaine** |  |  |  |  |
| No | 366/100.0 | 330/100.0 | 178/100.0 | 152/100.0 |
| Missing | 32/- | 28/- | 14/- | 14/- |

*^a^All participants including users and non-users of herbal medicines.*

| **Suppl. Table 2** Acute and chronic disorders reported by pregnant women during pregnancy. | | | | |
| --- | --- | --- | --- | --- |
|  | **Acute disorders** | | **Chronic disorders** | |
|  | **N** | **%^a^** | **N** | **%^a^** |
| Any disorder | 202 | 54.4 | 101 | 27.1 |
| No disorder | 169 | 45.6 | 272 | 72.9 |
| Missing | 27 | - | 25 | - |
| Gastroesophageal reflux | 69 | 18.6 | n.a. | n.a. |
| Iron deficiency/anaemia | 63 | 17.0 | n.a. | n.a. |
| Sickness/vomiting/hyperemesis gravidarum | 53 | 14.3 | n.a. | n.a. |
| Diabetes | 47 | 12.7 | 4 | 1.1 |
| Obstipation | 41 | 11.1 | n.a. | n.a. |
| Piles/haemorrhoids | 39 | 10.5 | n.a. | n.a. |
| Headache/migraine | 29 | 7.8 | 20 | 5.4 |
| Sleeping disorder | 27 | 7.3 | 1 | 0.3 |
| Allergies | 22 | 5.9 | 29 | 7.8 |
| Thyroid disorder | 21 | 5.7 | 24 | 6.4 |
| Diarrhoea | 21 | 5.7 | n.a. | n.a. |
| Varicose veins | 18 | 4.9 | n.a. | n.a. |
| Gastrointestinal tract disorder | 16 | 4.3 | 7 | 1.9 |
| Mood disorder | 13 | 3.5 | 5 | 1.3 |
| High blood pressure | 11 | 3.0 | 1 | 0.3 |
| Low blood pressure | 9 | 2.4 | 1 | 0.3 |
| Urinary tract infection | 9 | 2.4 | n.a. | n.a. |
| Asthma | 7 | 1.9 | 12 | 3.2 |
| Minor depression | 5 | 1.3 | 5 | 1.3 |
| Anxiety disorder | 3 | 0.8 | 3 | 0.8 |
| Heart disorder | 1 | 0.3 | 0 | 0.0 |
| Rheumatic disorder | 1 | 0.3 | 0 | 0.0 |
| Kidney disorder | 1 | 0.3 | 0 | 0.0 |
| Epilepsy | 0 | 0.0 | 2 | 0.5 |
| Major depression | 0 | 0.0 | 1 | 0.3 |
| Cancer | 0 | 0.0 | 1 | 0.3 |
| Others | 29 | 7.8 | 10 | 2.7 |

*^a^Percentage values without considering missing data.*

*Multiple answers were possible. Data are sorted by frequency of acute disorders. n.a., not asked.*

| **Suppl. Table 3** Reported symptoms during and before pregnancy. | | |  | |
| --- | --- | --- | --- | --- |
|  | **During pregnancy** | | **Before pregnancy** | |
|  | **N** | **%^a^** | **N** | **%^a^** |
| Fatigue | 294 | 79.5 | 84 | 22.7 |
| Nausea | 248 | 67.9 | 59 | 16.2 |
| Heartburn | 211 | 58.8 | 39 | 10.8 |
| Shortness of breath | 191 | 53.2 | 19 | 5.3 |
| Constipation | 166 | 48.3 | 89 | 25.9 |
| Insomnia | 145 | 42.9 | 50 | 14.8 |
| Vomiting | 128 | 38.3 | 63 | 19.0 |
| Dizziness | 96 | 29.3 | 55 | 16.7 |
| Headache | 95 | 28.4 | 122 | 36.5 |
| Oedema | 82 | 23.2 | 17 | 4.8 |
| Abdominal pain | 72 | 22.6 | 76 | 23.8 |
| High heart rate | 70 | 20.7 | 18 | 5.3 |
| Anxiety | 57 | 17.8 | 45 | 14.1 |
| Depressive mood | 33 | 9.9 | 43 | 12.8 |
| Others | 25 | 7.0 | 6 | 1.7 |

*^a^Percentage values without considering missing data.*

*Multiple answers were possible. Data are sorted by frequency of symptoms during pregnancy.*

| **Suppl. Table 4** Use of sedatives/anxiolytics and antidepressants during and before pregnancy. | | | | |
| --- | --- | --- | --- | --- |
|  | **During pregnancy** | | **Before pregnancy** | |
|  | **n/N** | **%^a^** | **n/N** | **% ^a^** |
| **Antidepressants** | 5/315 | 1.6 | 19/321 | 5.9 |
| Sertralin | 2/237 | 0.8 | 5/238 | 2.1 |
| Citalopram | 1/235 | 0.4 | 6/237 | 2.5 |
| Others | 2/240 | 0.8 | 9/242 | 3.7 |
| **Sedatives/anxiolytics** | 0/310 | 0.0 | 15/315 | 4.8 |
| Lorazepam | 0/242 | 0.0 | 11/243 | 4.5 |
| Diazepam | 0/243 | 0.0 | 3/243 | 1.2 |
| Others | 0/244 | 0.0 | 3/244 | 1.2 |

*^a^Percentage values without considering missing data.*
